# Skin whitening among Cameroonian female university students: knowledge, attitudes, practices and motivations

**DOI:** 10.1186/s12905-017-0385-z

**Published:** 2017-04-17

**Authors:** Emmanuel Armand Kouotou, Jobert Richie N. Nansseu, Hugues Adegbidi, Theophane Christel Joel Zoa Mebara, Elie Claude Ndjitoyap Ndam

**Affiliations:** 1 Biyem-Assi District Hospital, Yaoundé, Cameroon; 2 Faculty of Medicine and Biomedical Sciences, University of Yaoundé I, Yaoundé, Cameroon; 3 Yaoundé General Hospital, Yaoundé, Cameroon; 4 Sickle Cell Disease Unit, Mother and Child Centre, Chantal Biya Foundation, Yaoundé, Cameroon; 5 National University Teaching Hospital, Cotonou, Benin; 6 Department of Internal Medicine and Specialties, Faculty of Medicine and Biomedical Sciences, University of Yaoundé I, P.O. Box: 8314, Yaoundé, Cameroon

**Keywords:** Skin whitening, University student, Yaoundé, Cameroon

## Abstract

**Background:**

Lack of data on skin whitening (SW) among Cameroonian female university students prompted us to undertake the present study which aimed at assessing the knowledge, attitudes, practices and motivations of female university students vis-a-vis SW.

**Methods:**

This was a cross-sectional study conducted from January to April 2013 in 4 university campuses of Yaoundé, Cameroon. Any female student regularly registered in one of the study sites, who was present at the campus when the investigator visited and volunteered to participate in the study was enrolled.

**Results:**

Overall, we recruited 620 female students, their ages ranging from 16 to 46 years with a mean of 21.3 ± 2.9 years. Only 87 participants (14%) found that SW was a good practice. One hundred and sixty nine respondents (27.3%) were currently practicing SW with no age difference when compared to their counterparts (*p* = 0.09). The desire to have a uniform body skin color was the prevailing reason motivating the practice of SW (39.1%), followed by the need to have a soft skin (29%). Assessment of levels of knowledge regarding advantages of the black skin and deleterious effects of SW showed excellent scores (≥75% of good answers) only in 6.1 and 0.5% of cases respectively, with no difference between those practicing SW or not (all p values > 0.05).

**Conclusion:**

The practice of SW is common among Cameroonian female university students who should therefore be educated on the advantages of the black skin and the harmful effects of SW.

**Electronic supplementary material:**

The online version of this article (doi:10.1186/s12905-017-0385-z) contains supplementary material, which is available to authorized users.

## Background

The human skin color is one of the most perceptible phenotypic variations among humans, and is determined primarily by the type and amount of melanin synthesized within melanosomes as well as the pattern of melanosome distribution within the melanocytes. Seeking for a lighter skin tone has always drawn a major interest, as for centuries in Western societies, fair or light skin color has been a symbol of beauty, purity, sweetness, sex appeal, prominence, superiority and higher social ranking [1].

Skin whitening (SW) is the process consisting in the reduction in the physiological pigmentation of the body skin by the cosmetic use of bleaching agents [2]. Over time, this desire to appear “white” has become widespread; hence a global phenomenon reaching especially the sub-Saharan African part of the globe where a large majority of women is practicing SW, as the phenomenon is gradually getting rid of its shameful aspect [3]. In fact, in Dakar (Senegal), Diongue et al. found in 2013 that SW was practiced by 67% of the female population [4]. Besides, SW is also preponderant in other parts of the globe, especially in Asian countries such as Philippines [5]. The bleaching products used are diverse and increasingly sophisticated. Consequential complications, either local or systemic, can result from their chronic utilization and significantly impede the person’s health status and related quality of life [2, 3, 6–8].

In Cameroon and according to Kouotou et al., the practice of SW, also referred to as “stripping”, “make-up” or “mazembé”, is still a taboo, shameful and even reprehensible phenomenon [8]. In a previous report, we examined how SW is practiced among young women in Cameroon, and the composition of bleaching products used for this purpose [9]. However, opinions of women with regard to the practice of SW remain uninvestigated, as well as motivations of those who have become familiar with this practice. Willing to fil this gap, the present study was designed, aiming at assessing the knowledge, attitudes and practices of SW among young women living in Yaoundé (Cameroon), and motivations of those who practice SW.

## Methods

### Study design, setting and participants

We conducted a cross-sectional study between January and April 2013 in four university institutions of Yaoundé, the capital city of Cameroon, namely: the University of Yaoundé I, the University of Yaoundé II, the Siantou Higher Institute, and the Catholic University of Central Africa. These universities were randomly chosen among all the public and private university institutions located in the town.

During the study period, we consecutively and exhaustively recruited any female university student regularly registered in one of the just-cited study sites, regardless of her skin color, ethnicity, religion or social rank, who was present at the campus when the investigators visited and who voluntarily accepted to participate in the study. The minimal sample size was calculated, considering a 43.6% prevalence of SW reported by Kouotou et al. [8], and a 5% level of precision, hence a minimal number of 378 participants.

### Data collection

Before starting the study, we conceived an anonymous, structured and self-administered questionnaire for data collection, which was reviewed and validated by an expert committee of Cameroonian dermatologists who made sure that the questionnaire would permit to address all the objectives of the study. Subsequently, the questionnaire (Additional file 1) was pre-tested and amendments were made were necessary.

After clearly presenting all aspects of the study to each potential participant, we obtained a written and signed informed consent from participants (or their/parents/guardians) who accepted to take part in the study. Then, the participant was given the questionnaire to be filled, which was collected later on after the investigator has verified that the questionnaire has been adequately and completely filled.

Data were collected on socio-demographic background (age, level of education, marital status, and religion), opinions vis-à-vis SW, reasons underpinning this practice, and places of supplies in bleaching products. Besides, we assessed knowledge on the advantages of the black skin, (graded on 4 points), on the consequences of SW (graded on 18 points), and on the role of the dermatologist with regard to skin care (graded on 4 points). Each right answer was attributed a one point mark, whereas a wrong answer was given zero point. Subsequently, participants were grouped considering that their note was “low = L” (score <25%), “mediocre = M” (score 25–49.9%), “good = G” (score 50–74.9%) or “excellent = E” (score ≥ 75%).

### Statistical methods

Data were coded and analyzed using Epi info version 3.2.2 (Centre for Disease Control, Atlanta, USA). Results are presented as count (percentage) or mean ± standard deviation (SD) where appropriate. Comparison of qualitative variables used the Chi-square test or Fisher exact test, and the Student ***t*** test served for comparison of quantitative variables. A *p* value <0.05 was used to characterize statistically significant results.

### Ethical considerations

Prior to initiating the study, an ethical clearance was delivered by the Cameroon National Ethics Committee and authorizations were obtained from the institutional authorities of the different study sites. The study was conducted in conformity with the current revision of the Helsinki Declaration. Participants were informed about the various aspects and procedures relevant to the study, and were included after they have volunteered to take part in it. Each participant or his/her parent/guardian (for those aged < 18 years) provided a written and signed informed consent before enrolment in the study. Data collection was anonymous, and information was kept confidential.

## Results

One hundred and fifty five subjects were enrolled in each of the four study sites, giving a total of 620 participants. Ages ranged from 16 to 46 years, with a mean of 21.3 ± 2.9 years. The most represented age group was 21–25 years (Table 1). Only eighty seven participants (14%) judged SW to be a good practice. One hundred and sixty nine of our respondents (27.3%) were practicing SW, with no age difference when compared with those not practicing it (22.0 ± 3.4 vs. 21.1 ± 2.7, *p* = 0.09).

**Table 1:** Repartition of the study population according to age groups

Age groups (years)	Practice of skin whitening				Total	
	No		Yes			
	*N* = 451	%	*N* = 169	%	*N* = 620	(%)
15–20	213	34.3	57	9.2	270	43.6
21–25	216	34.8	91	14.6	307	49.5
26–30	20	3.2	19	3.1	39	6.3
31–35	1	0.2	1	0.2	2	0.3
>35	1	0.2	1	0.2	2	0.3

**Fig. 1:**
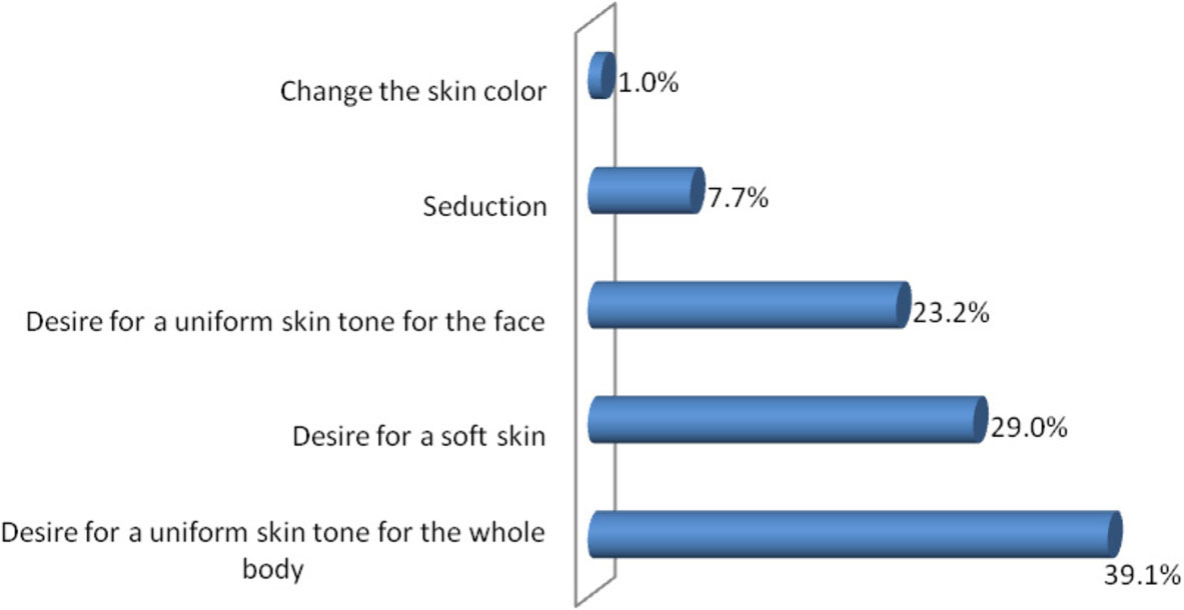
Motivations supporting the practice of skin whitening

The university students who were practicing SW were used to buying the bleaching products in non-specialized stores (72.4%), at the pharmacy (24.3%), from the aesthetician (17.8%), and 4.1% of them used homemade/craft compositions. Concerning the reasons motivating the practice of SW, the desire for a uniform skin tone was the prevailing reason (39.1%), followed by the desire for a soft skin (29%). The desire to change the skin color was the least encountered motivation (1.0%) (Fig. 1).

Table 2 spells out the level of knowledge with regard to advantages conferred by the black skin, consequences of SW and the role of the dermatologist towards skin care. Concerning the level of knowledge on the advantages of the black skin, excellent scores were obtained only in 7.7% (13/169) and 5.5% (25/451) of cases, respectively among those practicing SW in comparison to their counterparts, with no difference between the two groups (*p* = 0.380). Regarding the level of knowledge on the consequences of SW, excellent scores were recorded in 0.6% (1/169) and 0.4% (2/451) of cases among students using bleaching agents or not, respectively; the difference between the two groups was statistically non-significant (*p* = 0.438). Lastly, the role of the dermatologist was excellently known by nine students (5.3%) practicing SW in comparison to 37 students (8.2%) not practicing it, still with no statistical difference between the two groups (*p* = 0.218). Further results are presented elsewhere [9].

**Table 2:** Level of knowledge on the advantages of the black skin, the deleterious effects of skin whitening and the role of the dermatologist vis-à-vis skin care

		Practice of skin whitening				*p* value
Knowledge on		No		Yes		
	Note	*N* = 451	(%)	*N* = 169	(%)	
Advantages of the black skin						
	L	222	35.8	92	14.8	
	M	122	19.7	38	6.1	
	G	82	13.2	26	4.2	**0.380**
	E	25	4.0	13	2.1	
Role of the dermatologist						
	L	0	0.0	0	0.0	
	M	9	1.5	7	1.1	
	G	405	65.3	153	24.7	**0.218**
	E	37	6.0	9	1.5	
Negative effects of SW						
	L	178	28.7	79	12.7	
	M	215	34.7	70	11.3	
	G	56	9.0	19	3.1	**0.438**
	E	2	0.3	1	0.2	

*L* low, *M* medium, *G* good, *E* excellent, *SW* skin whitening

## Discussion

Results from the present study indicate that almost thirty percent of female university students (27.3%) were practicing SW, although only a few of them (14%) judged the practice to be a good one. The need to harmonize the skin tone of the whole body was the main reason supporting the practice of SW. Levels of knowledge relative to the advantages of the back skin and the consequences of SW were largely unsatisfactory. It appears therefore of great importance to educate our young female university students, and the whole population in general, on the tremendous benefits conferred by the black skin, and the deleterious effects of SW in terms of short-, medium-, and long-term complications. Furthermore, they should be counselled and encouraged to use adapted products to take care of their skin without irritating or aggressing it.

In our survey, ages ranged from 16 to 46 years (mean 21.3 ± 2.9 years), nearly concurring with reports from Gathse et al. where ages varied between 14 and 58 years (mean 26.6 years) [10]. The mean age of students using bleaching agents was 22.0 ± 3.3 years, comparable to results from Traoré et al. in Burkina Faso (mean 25 years) [11]. We could infer from our findings a precocious age at initiation of SW, this being explained perhaps by the fact that there is an increasing number of advertisements on bleaching agents which could influence the practice of young women.

Intriguingly, 64.5% of participants using lightening products recognized that the practice of SW was not a good one, contrasting thereby with the fact that they were currently practicing it. The same observation has been previously made by Mahé et al. in Dakar (Senegal) who found that 85% of their respondents practicing SW believed this was a harmful practice [12].

The willingness to harmonize the skin tone was the prevailing reason motivating the practice of SW, corroborating results from Dakar (Senegal) where the large majority of women practicing SW desired a uniform skin complexion [12]. Moreover, Mahé et al. found that 21% of their women wished to have a beautiful skin [12]. As the notion of beauty is relative depending on each individual, this may justify the choice of a particular bleaching product or composition rather than another one depending on its rapidity and power of action. Further studies dedicated at better ascertaining this concern are warranted in our milieu.

The level of knowledge with respect to the consequences of SW was very low in the majority of cases, with no difference between the users of bleaching agents when compared to their counterparts (*p* = 0.438). These findings indicate perhaps the absence of any relationship between knowledge and attitude or practice. In this regard, Petit et al. showed that although being aware of the risks incurred with the practice of SW, women were still engaged in this practice or did not want to stop it, as they were hardly prompted by the desire to become or remain beautiful [6]. Likewise, in a more recent South African survey, Dlova et al. observed that the large majority of women using bleaching products, regardless of their ethnicity (either Black or Indian) did not stop utilizing these products despite that they did not ignore consequential deleterious effects [13].

Remembering that in Cameroon the practice of SW remains a taboo and shameful phenomenon [8] and considering that information collected was only based on the participant’ declarations, it is therefore possible that this prevalence of SW practice we have obtained was perhaps underestimated. Another limitation of this study could lie in the fact that very few students accepted to participate in the study in comparison to the number of students registered in the different study sites, perhaps introducing some selection bias and precluding us from generalization of our results. Nonetheless, we randomly chose our study sites, and obtained a sample size greater than the minimum required.

## Conclusion

The cosmetic use of skin bleaching agents is a common practice among female university students residing in Yaoundé, Cameroon. Only few of them have judged SW to be a good practice. The desire to have a uniform body skin tone was the prevailing reason given to sustain the use of lightening cosmetics. These female students were less aware of the deleterious effects of SW. Interventions to educate these groups and the general population on the huge advantages conferred by the black skin and the potential harmful and irreversible effects of SW need urgent implementation in our milieu. Besides, they should be counselled and encouraged to use adapted products to take care of their skin without aggressing it.

## Additional file

Additional file 1: Questionnaire. (DOCX 35 kb)

## Abbreviations

SD: Standard deviation
SW: Skin whitening
