# Supplementary material for: Skin whitening among Cameroonian female university students: knowledge, attitudes, practices and motivations
Source: BMC Womens Health. 2017 Apr 17;17:33. doi: 10.1186/s12905-017-0385-z (PMC5392984; doi:10.1186/s12905-017-0385-z)
Supplement: Additional file 1: — Questionnaire. (DOCX 35 kb) [file 12905_2017_385_MOESM1_ESM.docx]

# QUESTIONNAIRE

| **Section 1: Identification** | | | |
| --- | --- | --- | --- |
| S1Q01 | Code | \|__\|__\|__\|__\| | |
| S1Q02 | Study site: 1 = The University of Yaoundé I; 2 = The University of Yaoundé II; 3 = The Siantou Higher Institute; 4 = The Catholic University of Central Africa | \|__\| | |
| S1Q03 | Age (years) | \|____\| | |
| S1Q04 | Origin/Ethnic group_______________________________________________________________________________________ | | |
| S1Q05 | Level of education: 1 = Never went to school; 2 = Primary school; 3 = Secondary school; 4 = University/college | | \|__\| |
| S1Q06 | Religion: 1 = Christian ; 2 = Muslim : 3 = Other (precise)______________________________________ | | \|__\| |
| S1Q07 | Residency ________________________________________________________________ | | |

| **Section 2: Knowledge and opinions** | | |
| --- | --- | --- |
| **Knowledge** | | |
| S2Q01 | What are the advantages of the black skin? | |
| S2Q01.1 | It protects against UV radiations better that other skin types  1 = Yes; 2 = No | \|__\| |
| S2Q01.2 | It is the most resistant one  1 = Yes; 2 = No | \|__\| |
| S2Q01.3 | It gets old less rapidly than other skin types  1 = Yes; 2 = No | \|__\| |
| S2Q01.4 | It is the least dry one  1 = Yes; 2 = No | \|__\| |
| S2Q02 | What is the role of the dermatologist? | |
| S2Q02.1 | He/she is the one who treats skin diseases: 1 = Yes ; 2 = No | \|__\| |
| S2Q02.2 | He/she is the one who takes care of the beauty and softness of the skin: 1 = Yes ; 2 = No | \|__\| |
| S2Q02.3 | He/she can take care of the beauty and softness of the skin:  1 = Yes ; 2 = No | \|__\| |
| S2Q02.4 | He/she can give advices on which products can be used to keep the skin beautiful and soft without aggressing it: 1 = Yes ; 2 = No | \|__\| |
| S2Q03 | What is the role of the aesthetician? | |
| S2Q03.1 | He/she is the one who treats skin diseases: 1 = Yes ; 2 = No | \|__\| |
| S2Q03.2 | He/she is the one who takes care of the beauty and softness of the skin: 1 = Yes ; 2 = No | \|__\| |
| S2Q03.3 | He/she can take care of the beauty and softness of the skin:  1 = Yes ; 2 = No | \|__\| |
| S2Q03.4 | He/she can give advices on which products can be used to keep the skin beautiful and soft without aggressing it: 1 = Yes ; 2 = No | \|__\| |
| S2Q04 | What is whitening the skin about? | |
| S2Q04.1 | Changing the skin color (from black to white): 1 = Yes; 2 = No | \|__\| |
| S2Q04.2 | Removing marks on the skin: 1 = Yes; 2 = No | \|__\| |
| S2Q04.3 | Cleaning in deep the skin: 1 = Yes; 2 = No | \|__\| |
| S2Q04.4 | Harmonizing the sin tone: 1 = Yes; 2 = No | \|__\| |
| S2Q04.5 | Making the skin more vivid and radiant: 1 = Yes; 2 = No | \|__\| |
| S2Q05 | What are the consequences of skin whitening? | |
| S2Q05.1 | Hyperpigmentation of cheekbones: 1 = Yes; 2 = No | \|__\| |
| S2Q05.2 | Hyperpigmentation of finger joints: 1 = Yes; 2 = No | \|__\| |
| S2Q05.3 | Infections: 1 = Yes; 2 = No | \|__\| |
| S2Q05.4 | Healing troubles: 1 = Yes; 2 = No | \|__\| |
| S2Q05.5 | Bleached skin marks: 1 = Yes; 2 = No | \|__\| |
| S2Q05.6 | Destruction of the skin layers: 1 = Yes; 2 = No | \|__\| |
| S2Q05.7 | Cancer: 1 = Yes; 2 = No | \|__\| |
| S2Q05.8 | Spots: 1 = Yes; 2 = No | \|__\| |
| S2Q05.9 | Hirsutism: 1 = Yes; 2 = No | \|__\| |
| S2Q05.10 | Stretchmarks: 1 = Yes; 2 = No | \|__\| |
| S2Q05.11 | Burnings: 1 = Yes; 2 = No | \|__\| |
| S2Q05.12 | Irritations: 1 = Yes; 2 = No | \|__\| |
| S2Q05.13 | Raised blood pressure: 1 = Yes; 2 = No | \|__\| |
| S2Q05.14 | Diabetes: 1 = Yes; 2 = No | \|__\| |
| S2Q05.15 | Body fat distribution disturbances: 1 = Yes; 2 = No | \|__\| |
| S2Q05.16 | Osteoporosis (fragility of bones): 1 = Yes; 2 = No | \|__\| |
| S2Q05.17 | Muscle weakness: 1 = Yes; 2 = No | \|__\| |
| S2Q05.18 | Obesity: 1 = Yes; 2 = No | \|__\| |
| **Opinions** | | |
| S2Q06 | What do you think are the characteristics of a beautiful skin? | |
| S2Q06.1 | Smooth: 1 = Yes; 2 = No | \|__\| |
| S2Q06.2 | Brilliant: 1 = Yes; 2 = No | \|__\| |
| S2Q06.3 | Light: 1 = Yes; 2 = No | \|__\| |
| S2Q06.4 | Without any wrinkle: 1 = Yes; 2 = No | \|__\| |
| S2Q06.5 | Without any mark: 1 = Yes; 2 = No | \|__\| |
| S2Q07 | Who encourages skin whitening? | |
| S2Q07.1 | The medias: 1 = Yes; 2 = No | \|__\| |
| S2Q07.2 | Relatives: 1 = Yes; 2 = No | \|__\| |
| S2Q07.3 | Friends/the entourage: 1 = Yes; 2 = No | \|__\| |
| S2Q07.4 | Men: 1 = Yes; 2 = No | \|__\| |
| S2Q08 | Do you think skin whitening is a good practice?  1 = Yes; 2 = No | \|__\| |

| **Section 3: Practices** | | |
| --- | --- | --- |
| S3Q01 | Do you use skin lightening products? 1 = Yes; 2 = No | \|__\| |
| S3Q02 | If Yes, what are your motivations? | |
| S3Q02.1 | To remove a precise skin mark: 1 = Yes; 2 = No | \|__\| |
| S3Q02.2 | To harmonize the skin tone of the face: 1 = Yes; 2 = No | \|__\| |
| S3Q02.3 | To harmonize the whole body skin tone: 1 = Yes; 2 = No | \|__\| |
| S3Q02.4 | To change the skin color: 1 = Yes; 2 = No | \|__\| |
| S3Q02.5 | To have a softer skin: 1 = Yes; 2 = No | \|__\| |
| S3Q02.6 | To seduce: 1 = Yes; 2 = No | \|__\| |
| S3Q03 | What is the rhythm of application of bleaching products?  1 = Every day; 2 = Two times weekly: 3 = Once in a month; 4 = Other (precise)__________________________________________ | \|__\| |
| S3Q04 | Since when have you been using these products?  1 = < 5 years; 2 = 5-10 years; 3 = > 10 years | \|__\| |
| S3Q05 | Has one of these products been prescribed by a dermatologist?  1 = Yes; 2 = No | \|__\| |
| S3Q06 | Have you already used one of the following products to lighten your skin? | |
| S3Q06.1 | Fruits (lemon, carrot…): 1 = Yes; 2 = No | \|__\| |
| S3Q06.2 | Bleach: 1 = Yes; 2 = No | \|__\| |
| S3Q06.3 | Antiseptic soap: 1 = Yes; 2 = No | \|__\| |
| S3Q06.4 | Straightener: 1 = Yes; 2 = No | \|__\| |
| S3Q06.5 | Soda crystals: 1 = Yes; 2 = No | \|__\| |
| S3Q06.6 | Toothpaste: 1 = Yes; 2 = No | \|__\| |
| S3Q06.7 | Fuel/kerosene: 1 = Yes; 2 = No | \|__\| |
| S3Q07 | Have you had a reaction? 1 = Yes; 2 = No | \|__\| |
| S3Q08 | If yes, which one? | |
| S3Q08.1 | Pruritus: 1 = Yes; 2 = No | \|__\| |
| S3Q08.2 | Irritation: 1 = Yes; 2 = No | \|__\| |
| S3Q08.3 | Burning: 1 = Yes; 2 = No | \|__\| |
| S3Q08.4 | Spot: 1 = Yes; 2 = No | \|__\| |
| S3Q09 | Where do you buy the whitening products?  1 = At the pharmacy; 2 = From the aesthetician; 3 = In non-specialized stores; 4 = Nowhere (homemade/craft compositions) | \|__\| |

Products used (table to be filled)

| Class | Type of product | Used: Yes (Y) ; No (N) | Name | Frequency of utilization |
| --- | --- | --- | --- | --- |
| Topic | Soap |  |  |  |
|  | Cream |  |  |  |
|  | Body milk |  |  |  |
|  | Body lotion |  |  |  |
| Injections |  |  |  |  |
